# Assessing brain function in stressed healthy individuals following the use of a combination of green tea, Rhodiola, magnesium, and B vitamins: an fMRI study

**DOI:** 10.3389/fnut.2023.1211321

**Published:** 2023-08-16

**Authors:** Gisèle Pickering, Lionel Noah, Bruno Pereira, Jonathan Goubayon, Vincent Leray, Ambre Touron, Nicolas Macian, Lise Bernard, Christian Dualé, Veronique Roux, Carine Chassain

**Affiliations:** ^1^Platform of Clinical Investigation Department, University Hospital Clermont-Ferrand, INSERM CIC, Clermont-Ferrand, France; ^2^Department of Pharmacology, University Clermont Auvergne, Inserm, Clermont-Ferrand, France; ^3^Sanofi, Gentilly, France; ^4^Clinical Research and Innovation Department, University Hospital Clermont-Ferrand, Clermont-Ferrand, France; ^5^Université Clermont Auvergne, Clermont Auvergne INP, CNRS, CHU Clermont Ferrand, ICCF, Clermont-Ferrand, France; ^6^Université Clermont Auvergne, CHU, CNRS, Clermont Auvergne INP, Institut Pascal, Clermont-Ferrand, France

**Keywords:** chronic stress, pain, magnesium, vitamins, green tea, L-theanine, Rhodiola, fMRI

## Abstract

**Introduction:**

This randomized, controlled, single-blinded trial assessed the effect of magnesium (Mg)-Teadiola (Mg, vitamins B6, B9, B12, Rhodiola, and green tea/L-theanine) versus placebo on the brain response to stressful thermal stimulus in chronically stressed, but otherwise healthy subjects. Impacts on stress-related quality-of-life parameters (depression, anxiety, sleep, and perception of pain) were also explored.

**Methods:**

The study recruited a total of 40 adults (20 per group), suffering from stress for more than 1 month and scaling ≥14 points on the Depression Anxiety Stress Scale (DASS)-42 questionnaire at the time of inclusion. Individuals received oral Mg-Teadiola or placebo for 28 days (D). fMRI analysis was used to visualize the interplay between stress and pain cerebral matrices, using thermal stress model, at baseline (D0) and after D28.

**Results:**

Based on blood-oxygen-level-dependent (BOLD) signal variations during the stress stimulation (before pain perception), a significantly increased activation between D0 and D28 was observed for left and right frontal area (*p* = 0.001 and *p* = 0.002, respectively), left and right anterior cingulate cortex (ACC) (*p* = 0.035 and *p* = 0.04, respectively), and left and right insula (*p* = 0.034 and *p* = 0.0402, respectively) in Mg-Teadiola versus placebo group. During thermal pain stimulation, a significantly diminished activation of the pain matrix was observed between D0 and D28, for left and right prefrontal area (both *p* = 0.001), left and right insula (*p* = 0.008 and *p* = 0.019, respectively), and left and right ventral striatum (both *p* = 0.001) was observed in Mg-Teadiola versus placebo group. These results reinforce the clinical observations, showing a perceived benefit of Mg-Teadiola on several parameters. After 1 month of treatment, DASS-42 stress score significantly decreased in Mg-Teadiola group [effect size (ES) −0.46 (−0.91; −0.01), *p* = 0.048]. Similar reductions were observed on D14 (*p* = 0.011) and D56 (*p* = 0.008). Sensitivity to cold also improved from D0 to D28 for Mg-Teadiola versus placebo [ES 0.47 (0.02; 0.92) *p* = 0.042].

**Conclusion:**

Supplementation with Mg-Teadiola reduced stress on D28 in chronically stressed but otherwise healthy individuals and modulated the stress and pain cerebral matrices during stressful thermal stimulus.

## 1. Introduction

Stress has an increasing prevalence in modern societies (1), reaching 29.6% in the general population (2), and its deleterious health consequences are numerous at individual and societal levels (3–8). Vitamins (e.g., vitamin B6) and herbal extracts such as green tea/L-theanine, Rhodiola, ashwagandha, and saffron extracts (9–11) have shown to possess stress– and anxiety-relieving effects (12–19). Previous clinical studies demonstrated that Mg-Teadiola (a combination of Mg; vitamins B6, B9, and B12; Rhodiola, and green tea extracts) significantly alleviated subjective stress and mood responses (20, 21) and increased spectral theta brain activity, thereby increasing the attentional capacity (22) under acute stress conditions in healthy individuals (20, 21). Further, a recent placebo-controlled, randomized clinical trial showed that Mg-Teadiola significantly decreased stress scores [using the validated Depression Anxiety Stress Scale (DASS)-42 questionnaire] following 14 and 28 days of treatment in stressed but otherwise healthy individuals. Interestingly, a significant reduction in sensitivity to cold pain and a trend for lower sensitivity to warm pain was also reported (23). The way Mg-Teadiola or its components modulate both stress and pain is yet to be explored.

The relationship between stress and perception of pain has been reported earlier (24). Chronic stress and pain share neural circuits that operate in the amygdala, the hippocampus, and the prefrontal cortex (PFC) (25), and cortisol plays an essential role in the PFC-dependent regulation of the hypothalamic–pituitary–adrenal (HPA) axis and in the processing of emotion-related information by the amygdala (26). Usually, the response toward the similar psychosocial stressors or similar pain intensity varies significantly among different individuals based on differences in their executive functional capacities, self-control, and self-regulation. Executive functions that are generated in PFC are functionally connected to the limbic system and are regulated by the hippocampal-PFC pathway. This pathway is also essential for emotional regulation, which is also vulnerable to dysregulation by chronic stress and pain. Though a short-term stress is generally adaptive, a repeated or continued exposure to psychosocial stressors may lead to the impairment of the mechanisms of self-regulation and self-control, resulting in maladaptive behavior (e.g., vulnerability to adverse emotional and stress experience and exaggerated pain perception) (24).

Functional neuroimaging technique [functional magnetic resonance imaging (fMRI)] (27, 28) represents an important tool to investigate the beneficial impact of Mg-Teadiola at the brain level as it is the primary organ involved in the interplay between stress and pain pathways. fMRI is a noninvasive imaging technique that can be used for mapping brain cerebral areas, neural circuits, and networks critical to the stress response and also to pain integration. The fMRI technique allows to study the stress response in human brains (29). Given the importance of executive functioning in the stress–pain interplay, and because it is impaired under stress, the true efficacy of executive functioning should be evaluated under stressful conditions (30). For this, several paradigms have been developed to induce acute stress in fMRI settings (29), and these underline the complexity of activation of brain areas involved in emotion and endocrine response. Anticipation of the fMRI environment itself may serve as a stressful variable experience (31). Concerning the brain signature of pain (32, 33), the context and emotional status of the individual can influence pain perception, and reciprocal neural, endocrine, and immune interactions exist between pain and stress (34).

The primary study demonstrated the beneficial effects of Mg-Teadiola versus placebo on stress and pain perception in chronically stressed, otherwise healthy individuals (23). This ancillary study evaluated—with fMRI—the cerebral effect of Mg-Teadiola versus placebo in stressed but otherwise healthy individuals and further explored their neural activity when exposed to an acute physical stress (thermal stimulus).

## 2. Materials and methods

### 2.1. Study setting

This section has been previously described in a recent article (23). This was a 28-day, randomized, single-blinded, placebo-controlled, parallel-group study. The study was conducted at the Platform of Clinical Investigation Center, University Hospital, Clermont-Ferrand, France from July 2020 to July 2021.

**Figure 1:**
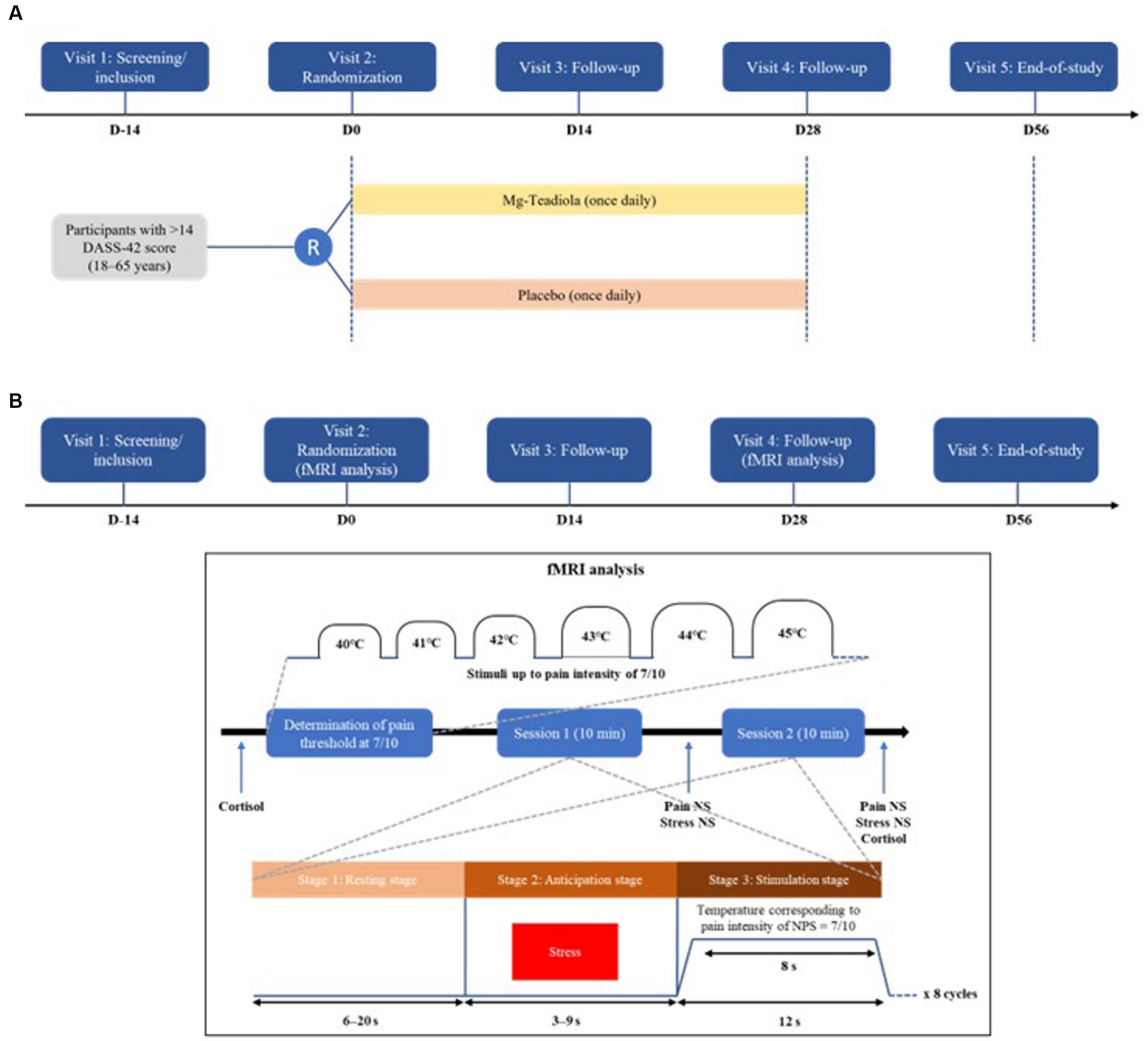
Study design and thermal pain stimulus cycles **(A)** Study design **(B)** Thermal pain stimulus cycles Mg-Teadiola, a combination of 150 mg magnesium (Mg), 0.7 mg vitamin B6, 0.1 mg vitamin B9, 1.25 μg vitamin B12, 222 mg of Rhodiola extract, and 125 mg of green tea extract including 50 mg of L-theanine. D, day; DASS-42, Depression Anxiety Stress Scale; Mg, magnesium; NPS, Neuropathic Pain Scale; NS, numerical scale.

The study was organized in five visits (Figures 1A,B): inclusion visit [visit 1, 14 days (D) before visit 2 (D0–14)], randomization visit [D0 (visit 2)], two follow-up visits [D14 (visit 3) and D28 (visit 4)], and end-of-study visit [D56 (visit 5)].

### 2.2. Individuals

All individuals were screened from the volunteer database of the Platform of Clinical Investigation Center, University Hospital, Inserm 1,405 in Clermont-Ferrand, France. This is an ancillary study of a primary study by Noah et al. (23) wherein 40 volunteers participated in the fMRI study (23).

The individuals gave their informed consent before participation in the study. Individuals were men or women between 18 and 65 years of age. At the time of inclusion, individuals reporting stress for at least 1 month, scaling greater than or equal to 14 on the DASS-42 stress questionnaire, and not ready for introducing any new treatment, diet, or use of any new analgesic and anti-inflammatory drugs, were included in the study. Individuals who were contraindicated to performing MRI (i.e., having claustrophobia, hearing aid, pacemaker, or brain clip), contraindicated in taking Mg (a plasma Mg >1.07 mmol/L), with moderate or severe kidney failure, with an inability to distinguish colors during the MRI and an inability to distinguish sensation resulting from nociceptive stimuli during psychometric tests, were excluded from the study. All the inclusion and exclusion criteria are detailed in the Supplementary Information 1.

### 2.3. Randomization, masking, and treatment

Details of this section have been reported earlier (23). Briefly, each participant received orally either Mg-Teadiola or placebo treatment, once a day in the morning for 28 days. The attribution of treatment between the two arms was equal (1:1). Mg-Teadiola tablet is composed of 150 mg of elemental Mg, 0.7 mg of vitamin B6, 0.1 mg of vitamin B9, 1.25 μg of vitamin B12, 222 mg of Rhodiola (*Rhodiola rosea* L.) extract, and 125 mg of green tea (*Camellia sinensis* [L.] Kuntze) extract (containing 50 mg of L-theanine) (35). This product was provided by Sanofi-Aventis Group. Placebo was a tablet composed of only excipients.

The allocation of treatments followed a predefined randomization plan and was performed by an individual who did not contribute to the study protocol. To avoid bias, a randomization list was placed in a sealed envelope and was blinded from the individuals, investigator, and other personnel involved in this study. Although the placebo and drug were distinguishable by appearance, the identity of placebo and drug was not revealed to the patients. The randomization code was computer-generated through randomly permuted blocks. Within each block, the number of participants allocated to each of the two treatment arms was equal (23).

#### 2.3.1. Study objectives

The primary objective of the parent study was to demonstrate the efficacy of Mg-Teadiola in managing stress in healthy individuals (23). This ancillary study evaluated the effect of Mg-Teadiola on brain areas activation related to stress (thermal stimulus) in healthy volunteers after 28 days of use. Other objectives were the evaluation of the impact of supplementation on stress, anxiety, depression, and sleep and its effects on biological parameters such as the levels of salivary cortisol and Mg levels in plasma, erythrocyte, and urine in the volunteers selected for the study.

### 2.4. Endpoints

The details of the primary endpoint (change in the DASS-42 stress scores from baseline to D28) and secondary endpoints (change in the stress, anxiety, and depression scores from baseline to D14 and/or D56 using DASS-42), of the parent study have been reported earlier (23). In the present ancillary study, main endpoint was the changes in cerebral areas (stress matrix, pain matrix) activation related to stress during the fMRI between baseline (D0) and D28 with Mg-Teadiola as compared with placebo.

Other endpoints included (i) the change in the DASS-42 stress subscale scores from D0 to D14, D28, and D56; (ii) the change in the DASS-42 anxiety subscale scores from D0 to D14, D28, and D56; (iii) the change in the DASS-42 depression subscale scores from D0 to D14, D28, and D56; (iv) the change in the Pain Catastrophizing Scale (PCS) scores from D0 to D14, D28, and D56; (v) the change in the Pittsburg Sleep Quality Index (PSQI) scores from D0 to D14, D28, and D56; (vi) the change in the quantitative somatosensory thermotest (QST) temperature from D0 to D28; (vii) the change in levels of salivary cortisol from D0 to D28; (viii) the change in levels of Mg in blood and urine from baseline to D14, D28, and D56; and (ix) the frequency of adverse events (AEs).

### 2.5. Study measurements

#### 2.5.1. Functional magnetic resonance imaging

Experiments were conducted on a Siemens Magnetom Vida 3.0 T using a 64-channel head coil, with individuals lying supine. Initial localizer images were acquired in three planes as a reference for slice positioning for subsequent fMRI studies. A standard whole-brain gradient echo planar imaging sequence was used for the functional scans (repetition time = 2,000 ms; echo time = 30 ms; 300 volumes field of view = 192 × 192 mm^2^, matrix = 128 × 128, voxel size = 2.0 × 2.0 × 2.0 mm^3^, simultaneous multislice (SMS) accelerator factor 2, acquisition time = 10 min). Two functional runs were acquired during thermal stimulation. A high-resolution volumetric three-dimensional (3D) T1-weighted acquisition was performed—for anatomical overlay of activation—in the same session as the functional scans (magnetization-prepared rapid acquisition gradient echo [MPRAGE] sequence with repetition time = 1,800 ms, echo time = 2.46 ms, inversion time = 900 ms, field of view = 240 × 240 mm^2^, matrix = 512 × 512, voxel size = 0.4 × 0.4 × 0.9 mm^3^, acquisition time = 3 min 38 s). To achieve synchronization, the trigger output of the scanner was used to initialize the fMRI paradigm and triggers from contact heat-evoked potential stimulation and the scanner were recorded together. fMRI sequences were assessed in the following order: anatomical scout, 3D brain volume, fMRI blood-oxygen-level-dependent (BOLD) sequences echo planar imaging (thermal stimulation).

#### 2.5.2. Evoked thermal pain stimulation

During fMRI sessions, volunteers were comfortably lying on the examination table of the MRI scanner with the thermode strapped on the dominant hand for thermal tests. First, the pain threshold—defined as the temperature where the stimulation becomes painful—was determined after an increase of temperature until pain reached 7 out of 10 on the numerical scale (Figures 1A,B). The stimulation paradigm consisted of eight cycles followed by 6–20 s rest followed by an anticipation visual stress challenge before pain stimuli. During stress challenge (3–9 s), the temperature of the thermode was 32°C and a red color was presented to individuals followed immediately by pain stimuli. Stimuli at the noxious stimulation temperature lasted 8 s. The cerebral “stress” matrix and “pain” matrix were identified using the images acquired during these two respective sequences (i.e., stress challenge, then pain stimulus). fMRI BOLD acquisitions were repeated twice for a global duration of 20 min.

#### 2.5.3. The Depression Anxiety Stress Scale-42 questionnaire

This questionnaire is an auto-evaluation of 42 items assessing the negative emotional states over the last week. It allows the evaluation of the mental health owing to 3 scales composed of 14 items: depression, anxiety, and stress (36, 37). For each question, individuals were graded between 0 “not present” and 3 “very frequently present”. Stress and pain scores were categorized as 0–14 “normal”, 15–18 “mild”, 19–25 “moderate”, 26–33 “severe”, or 34+ “extremely severe”.

#### 2.5.4. Quantitative somatosensory thermotest

Quantitative somatosensory thermotest (38) with an advanced thermal stimulator thermode connected to a Medoc PATHWAY (Medoc Ltd., Ramat Yishay, Israel) was used to apply thermal stimuli. The stimuli were delivered to the thenar eminence of the dominant hand from a D0 value of 32°C, and the chosen paradigm was applied. QST testing included thermal detection and thermal pain thresholds: cold detection and warm detection thresholds and cold pain and warm pain thresholds. From the D0 value of 32°C, the Medoc PATHWAY delivered an adjustable temperature peak (in cold and heat, depending upon a regular slope of 1°C) and was controlled by rapid feedback. This device was used to evaluate the thermal detection (when the individual began to feel the thermal change) and pain threshold (when the individual began to feel pain) to heat and cold by calculating the mean of three measures.

#### 2.5.5. Pain catastrophizing scale

The PCS is an evaluation of 13 items in which the individuals describe how they feel during a pain experience (39). It allows to assess the type of thought and emotion related to pain. For each question, individuals were graded between 0 “not at all” and 4 “all the time”. The final score was a sum of the scores for each question. A high value represented a higher catastrophism.

#### 2.5.6. The Pittsburg Sleep Quality Index

The PSQI is an evaluation of 19 items measuring sleep quality during the last month (40). It allows to assess seven parameters: subjective sleep quality, sleep latency, sleep duration, usual sleep efficiency, sleep disorders, use of a sleep medication, and poor daytime fitness. The global score was a sum of these domains and varied from 0–21. A high score represented a higher alteration of the quality of sleep.

#### 2.5.7. Salivary collection

Salivary collection was done before D0 and after fMRI (D28) to assess free cortisol, and the change in the salivary cortisol concentration from D0 to D28 was determined using a radioimmunoassay (Gamma Coat, DiaSorin, Stillwater, MN, United States) with a modified procedure after dichloromethane extraction as previously described (41). The limit of quantification was 2 nmol/L. The intra-assay coefficients of variation (CVs) were 7.1 and 3.0% at 10.1 and 22.6 nmol/L, respectively. The inter-assay CVs were 12.7 and 10.2% at 8.7 and 48.0 nmol/L, respectively.

#### 2.5.8. Magnesium assays

Magnesium assays were assessed by taking a total of 40 mL of venous blood from each patient and collected into heparin tube with gel (for serum Mg) or a heparin tube without gel (for erythrocyte Mg). Urinary Mg levels were measured and collected for 24 h, a day before visits 2, 3, 4, and 5. The tubes were sent to the biochemistry department of the University Hospital of Clermont-Ferrand for assay. Urinary and serum Mg levels were analyzed using the Dimension Vista® System Flex® Mg reagent cartridge (MAGNETOM Vida from Siemens). This is a modified version of the methyl thymol blue complexometric procedure. Methyl thymol blue forms a blue complex in the presence of Mg, and the amount of complex formed can be measured using a bichromatic endpoint method to determine the concentration of Mg. Erythrocyte Mg concentration was measured using a colorimetric method based on the formation of a colored complex of Mg with Xylidyl blue reagent in alkaline solution (Eurofins Biomnis, Lyon, France) (42).

#### 2.5.9. Safety

All AEs were reported and analyzed for each visit according to the intensity, duration, and evolution to determine if the AEs were due to the study combination.

## 3. Statistical analysis

### 3.1. Sample size calculation

A sample size estimation has been reported in the primary study (23). As this was an ancillary study, no sample estimation was assessed. However, according to (i) previous results reported in the literature; (ii) CONSORT 2010 statement, extension to randomized pilot and feasibility trials; and (iii) Cohen’s recommendations, which define effect-size bounds as small (effect size [ES]: 0.2), medium (ES: 0.5), and large (ES: 0.8, “grossly perceptible and therefore large”), we estimated that 20 individuals per arm would allow to fulfill the primary objective of this study with a satisfactory statistical power as sample sizes of 40 are generally able to detect regions with high ESs (43).

### 3.2. Statistical methods

#### 3.2.1. Functional magnetic resonance imaging data analysis

The fMRI data were preprocessed and analyzed using the Statistical Parametric Mapping software (44) on MATLAB R2018b (Mathworks Inc., Natick, MA, United States). The anatomical scan was spatially normalized to the avg152 T1-weighted brain template defined by the Montreal Neurological Institute using the default parameters (nonlinear transformation). Functional volumes were temporally and spatially realigned and normalized (using the combination of deformation field, co-registered structural and sliced functional images) and smoothed with an isotropic Gaussian kernel (full-width at half-maximum = 6 mm).

For each participant and each time point (D0 and D28), first-level statistical parametric maps (SPMs) were first generated using the general linear model to describe the variability of the data on a voxel-by-voxel basis. The model consisted of a boxcar function, using the stress-related and rest-related blocks for stress stimulation and the pain-related and rest-related blocks for pain stimulation as regressors of interest, convolved with the canonical SPM hemodynamic response function. The six motion regressors (translation and rotation) were also included in the model. Functional data were filtered with a 128-s high-pass filter. The contrasts between stimulations and rest were then generated.

Statistical parametric maps for each experimental factor and each participant calculated at the first level were then entered in a second-level two-factor analysis of variance (ANOVA) (group factor and time-point factor with repeated measure). Then, BOLD response amplitude for stress and pain stimulation were assessed for a significant decrease between D28 and D0 sessions and between the two groups—placebo and Mg-Teadiola. All statistical comparisons were performed with a voxel wise threshold of *p* < 0.05 and a cluster extent threshold of five voxels.

For a given zone of interest and based on previous ANOVA statistical results, we extracted each participant’s percent signal changes with the contrast stimulation-rest at D0 higher than stimulation-rest at D28 for the two groups using MarsBaR software (45). This beta analysis allowed to test for the first time a correlation between the BOLD signal variation between D28 and D0 (D0 > D28) and the difference in the DASS-42 stress subscale scores between D28 and D0 for both Mg-Teadiola and placebo groups.

#### 3.2.2. Clinical data analysis

Continuous data were expressed as mean and standard deviation (SD). The Shapiro–Wilk test was used to analyze the assumption of the Gaussian distribution. The comparisons between Mg-Teadiola and placebo groups at D28 were performed using the analysis of covariance adjusted on D0 value of the dependent endpoint. The results were expressed using ES and 95% confidence interval (CI), and the clinically relevant difference was defined based on judgment of expert clinician. When appropriate, a logarithmic transformation of the dependent variables was proposed. Similarly, the comparisons between randomization groups at D14 and D56 were carried out. No adjustments were made for multiple comparisons. Because of the potential for type I error due to multiple comparisons, the findings for analyses of secondary analyses were interpreted as exploratory with no adjustment made for multiple comparisons. Statistical analysis was performed with Stata software (version 15, Stata Corp., College Station, TX, United States).

## 4. Results

### 4.1. Individuals and baseline characteristics

**Figure 2:**
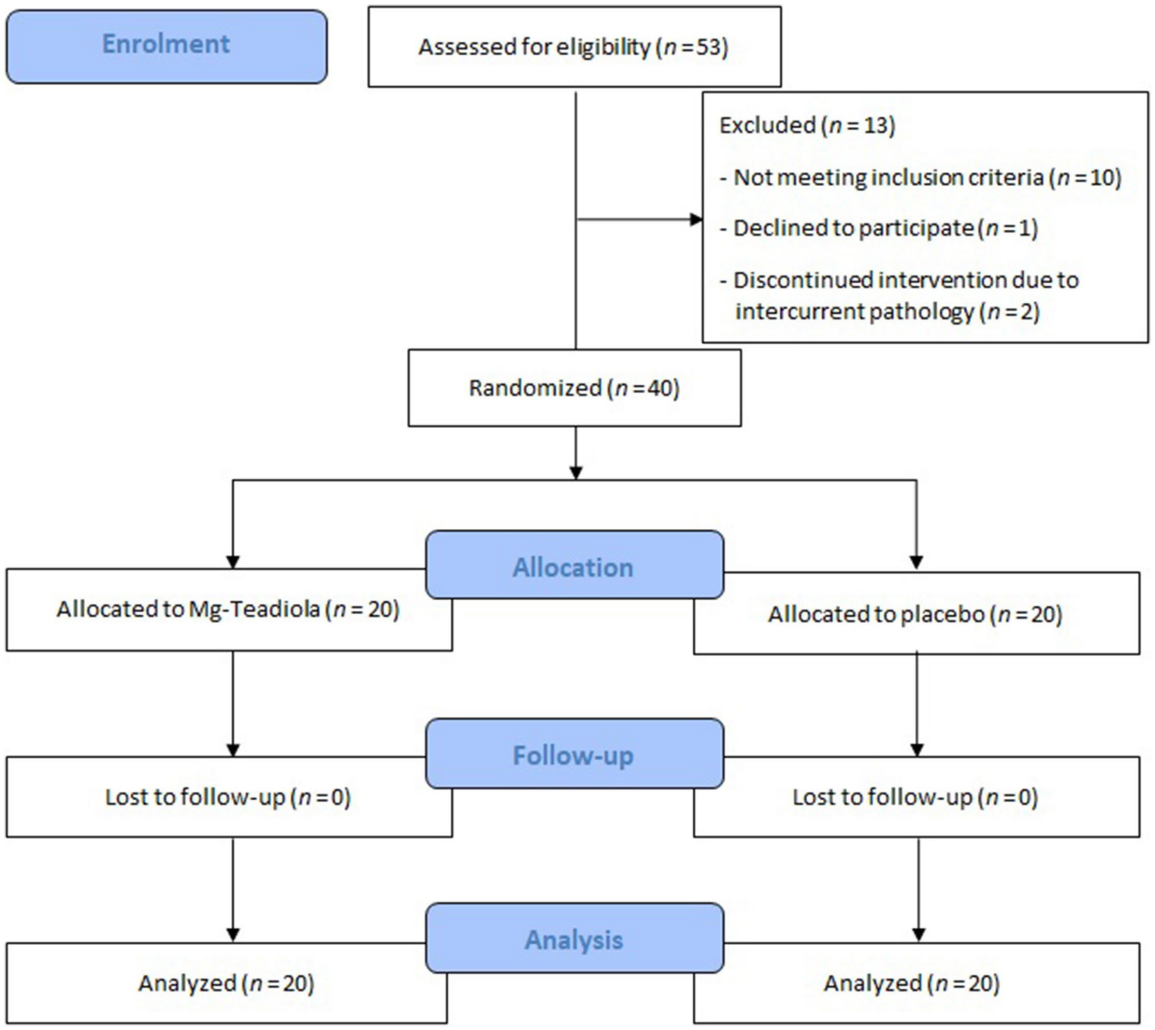
Patient disposition Mg-Teadiola, combination of 150 mg Mg, 0.7 mg vitamin B6, 0.1 mg vitamin B9, 1.25 μg vitamin B12, 222 mg of Rhodiola extract, and 125 mg of green tea extract including 50 mg of L-theanine. Mg, magnesium.

Out of 53 individuals screened, 40 were randomized (Mg-Teadiola: *n* = 20; placebo: *n* = 20, Figure 2), and all individuals completed the study. Overall, baseline characteristics (Table 1) were comparable between the two groups. Median age was 22.9 ± 4.4 years in the Mg-Teadiola group and 23.7 ± 6.5 years in the placebo group. Mean body mass indices (Mg-Teadiola: 22.5 ± 4.3; placebo: 22.9 ± 3.8) as well as psychological profiles were comparable in both groups. No relevant psychological disturbances were observed.

**Table 1:** Depression Anxiety Stress Scale (DASS)-42, quantitative somatosensory thermotest (QST), and biological measures following supplementation with Mg-Teadiola versus placebo at visit 2 (baseline, D0) and visit 4 (D28).

	D0		D28		
	Placebo *n* = 20	Mg-Teadiola *n* = 20	Placebo *n* = 20	Mg-Teadiola *n* = 20	ES (95% CI) *value of p*
**DASS-42**					
DASS stress	29.6 ± 7.8	24.4 ± 6.1	21.8 ± 9.7	16.8 ± 5.4	**−0.46 [−0.91; −0.01] p = 0.048**
DASS anxiety	17.4 ± 7.8	12.9 ± 8.1	12.9 ± 8.3	7.9 ± 5.6	−0.29 [−0.74; 0.16] *p* = 0.201
DASS depression	11.5 ± 9.4	9.9 ± 7.7	6.8 ± 6.8	4.2 ± 4.0	−0.33 [−0.78; 0.13] *p* = 0.154
**PCS-Catastrophism**	18.9 ± 11.6	13.3 ± 8.3	14.6 ± 11.7	9.1 ± 7.5	−0.13 [−0.59; 0.32] *p* = 0.554
Rumination	6.6 ± 4.7	4.7 ± 3.2	4.9 ± 4.5	3.0 ± 2.4	−0.19 [−0.65; 0.26] *p* = 0.396
Magnification	4.7 ± 3.2	4.1 ± 3.1	4.2 ± 3.0	2.3 ± 2.2	**−0.60 [−1.05; −0.15] p = 0.011**
Helplessness	7.7 ± 5.8	4.6 ± 3.7	5.5 ± 5.8	3.8 ± 3.5	0.04 [−0.42; 0.49] *p* = 0.864
**PSQI-Sleep**	8.7 ± 2.6	7.0 ± 2.4	7.1 ± 3.1	5.1 ± 2.0	−0.36 [−0.81; 0.10] *p* = 0.119
Subjective sleep quality	1.8 ± 0.6	1.6 ± 0.7	1.6 ± 0.7	1.3 ± 0.7	−0.25 [−0.71; 0.20] *p* = 0.263
Sleep latency	2.2 ± 1.0	2.0 ± 0.9	1.8 ± 1.2	1.3 ± 0.8	−0.30 [−0.75; 0.15] *p* = 0.188
Sleep duration	0.7 ± 0.9	0.6 ± 0.8	0.5 ± 0.7	0.4 ± 0.8	−0.05 [−0.50; 0.40] *p* = 0.826
Habitual sleep efficiency	0.5 ± 0.8	0.2 ± 0.4	0.4 ± 0.7	0.2 ± 0.5	−0.19 [−0.64; 0.26] *p* = 0.403
Sleep disturbances	1.7 ± 0.5	1.3 ± 0.4	1.4 ± 0.5	1.1 ± 0.2	−0.34 [−0.80; 0.11] *p* = 0.132
Use of sleeping medication	0.3 ± 0.8	0.1 ± 0.3	0.2 ± 0.5	0.1 ± 0.2	−0.08 [−0.54; 0.37] *p* = 0.713
Daytime dysfunction	1.6 ± 0.7	1.3 ± 0.5	1.3 ± 0.8	0.8 ± 0.6	−0.29 [−0.74; 0.16] *p* = 0.199
**QST (°C)**					
QST warm sensibility	33.5 ± 0.5	33.6 ± 0.8	33.9 ± 0.8	34.0 ± 0.9	0.01 [−0.44; 0.47] *p* = 0.959
QST cold sensibility	31.1 ± 0.5	30.9 ± 1.0	30.0 ± 1.5	30.6 ± 1.0	**0.47 [0.02; 0.92] p = 0.042**
QST warm pain	43.0 ± 2.8	41.9 ± 4.3	43.0 ± 2.6	42.4 ± 3.5	−0.01 [−0.46; 0.45] *p* = 0.974
QST cold pain	16.2 ± 10.0	16.3 ± 11.4	17.9 ± 9.6	15.2 ± 10.8	−0.27 [−0.72; 0.18] *p* = 0.236
**Biological measures**					
Plasma Mg (mmol/L)	0.85 ± 0.06	0.86 ± 0.09	0.89 ± 0.06	0.88 ± 0.05	−0.07 [−0.67; 0.54] *p* = 0.828
Urinary Mg (mmol/24 h)	3.45 ± 1.49	3.89 ± 1.58	3.46 ± 1.57	3.98 ± 1.53	0.33 [−0.28; 0.94] *p* = 0.291
Salivary cortisol (ng/mL)	−0.54 ± 0.75	−0.46 ± 1.24	−0.28 ± 0.43	−0.31 ± 0.91	−0.01 [−0.46; 0.44] *p* = 0.965

Values are mean ± SD unless otherwise stated. Significant *p*-values are in bold.

CI, confidence interval; D, day; DASS-42, Depression Anxiety Stress Scale; ES, effect size; Mg, magnesium; Mg-Teadiola, combination of Mg, vitamins B6, B9, B12, Rhodiola, and green tea/L-theanine; PCS Pain Catastrophizing Scale; PSQI, Pittsburg Sleep Quality Index; QST, quantitative somatosensory thermotest; SD, standard deviation.

### 4.2. Functional magnetic resonance imaging outcomes

**Figure 3:**
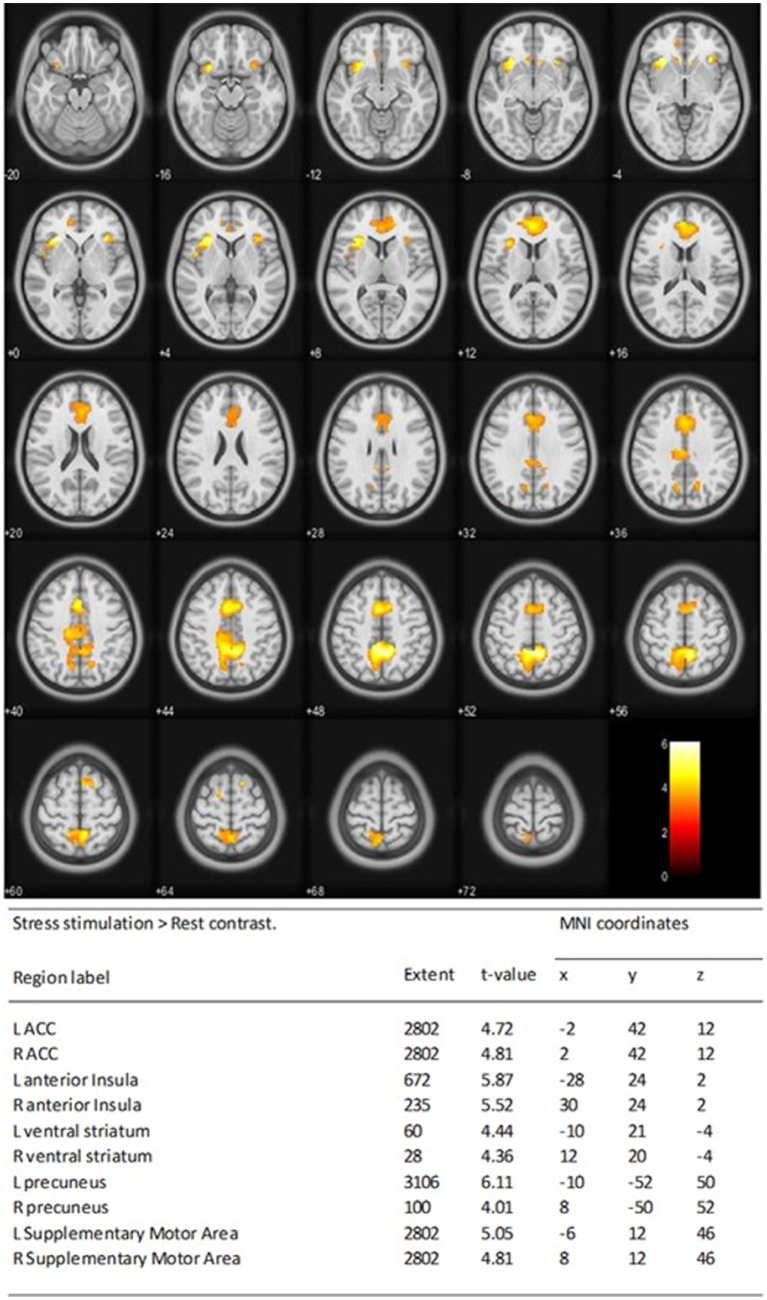
Stress matrix at D0 showing activation of the left (L) and right (R) of anterior cingulate cortex (ACC), the L and R of anterior insula (AI), the L and R of ventral striatum, the L and R of precuneus, and supplementary motor cortices One-sample *t*-test statistical results of the whole-brain analysis of the BOLD responses of stress stimulation > rest contrast at D0 for all individuals was performed. Statistical *t* maps were overlaid on MNI slices using a voxel-wise threshold of *p* < 0.001 and an extent threshold of 20 voxels. Regions were automatically labeled using the Anatomy Toolbox atlas of SPM and were presented in the table below. *x*, *y*, and *z* were MNI coordinates in the L-R, anterior–posterior, and inferior–superior dimensions, respectively. All peaks were significant at a voxel-wise threshold of *p* < 0.001 (extend threshold = 20 voxels). ACC, anterior cingulate cortex; AI, anterior insula; BOLD, blood oxygen level dependent; L, left; R, right; MNI, Montreal Neurological Institute; SPM, Statistical Parametric Mapping.

**Figure 4:**
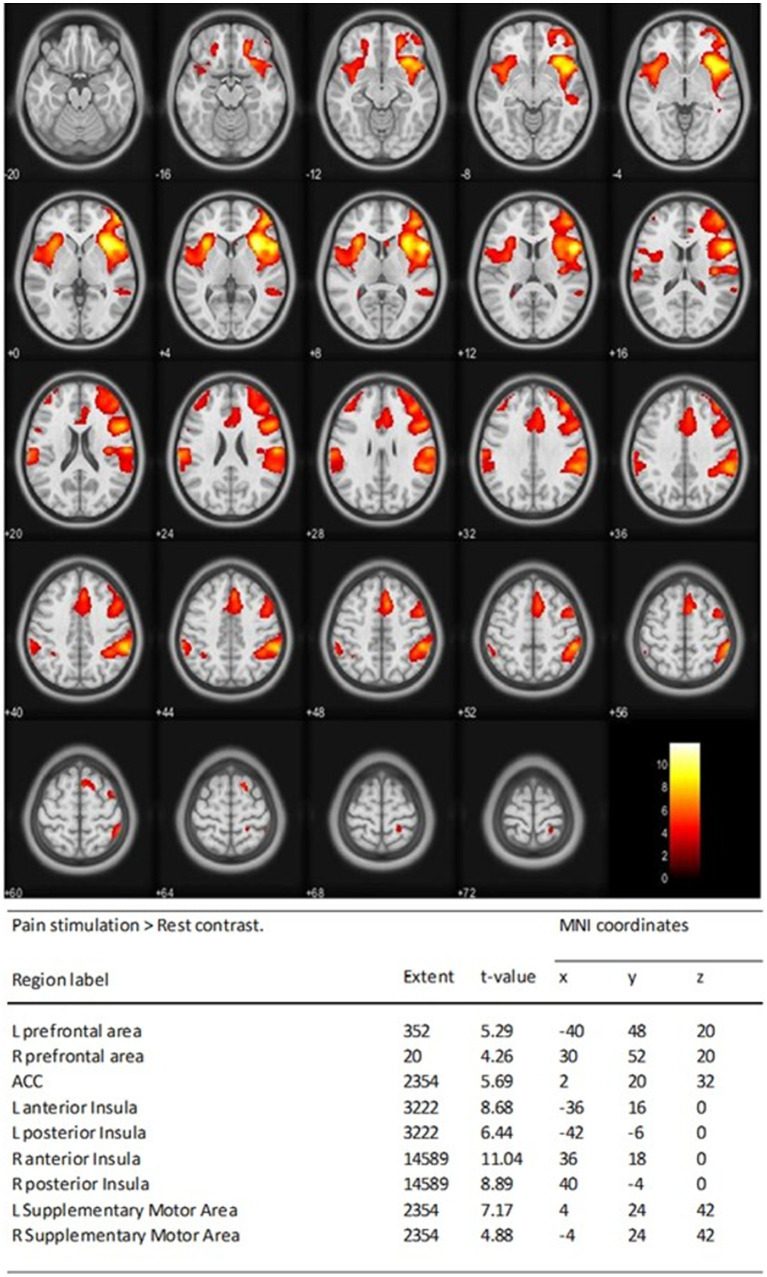
Pain matrix at D0 showing activation of the L and R of prefrontal cortices, ACC, anterior and posterior, the L and R of insula, and supplementary motor cortices One-sample *t*-test statistical results of the whole-brain analysis of the BOLD responses of pain stimulation > rest contrast at D0 for all individuals was performed. Statistical *t* maps are overlaid on MNI slices using a voxel-wise threshold of *p* < 0.001 and an extent threshold of 20 voxels. Regions were automatically labeled using the Anatomy Toolbox atlas of SPM and were presented in the table below. *x*, *y*, and *z* were MNI coordinates in the L-R, anterior–posterior, and inferior–superior dimensions, respectively. All peaks were significant at a voxel-wise threshold of *p* < 0.001 (extend threshold = 20 voxels). ACC, anterior cingulate cortex; BOLD, blood oxygen level dependent; L, left; R, right; MNI, Montreal Neurological Institute; SPM, Statistical Parametric Mapping.

At D0, during acute stress stimulation for all individuals, robust BOLD signals were seen in cortical regions, mainly the left and right occipital cortices. In addition, during acute pain stimulation, robust BOLD signals were seen in cortical regions typically involved in pain processing. These areas included the prefrontal cortices, anterior cingulate cortex (ACC) (anterior, medium, and dorsal), and insula (Figures 3, 4).

**Figure 5:**
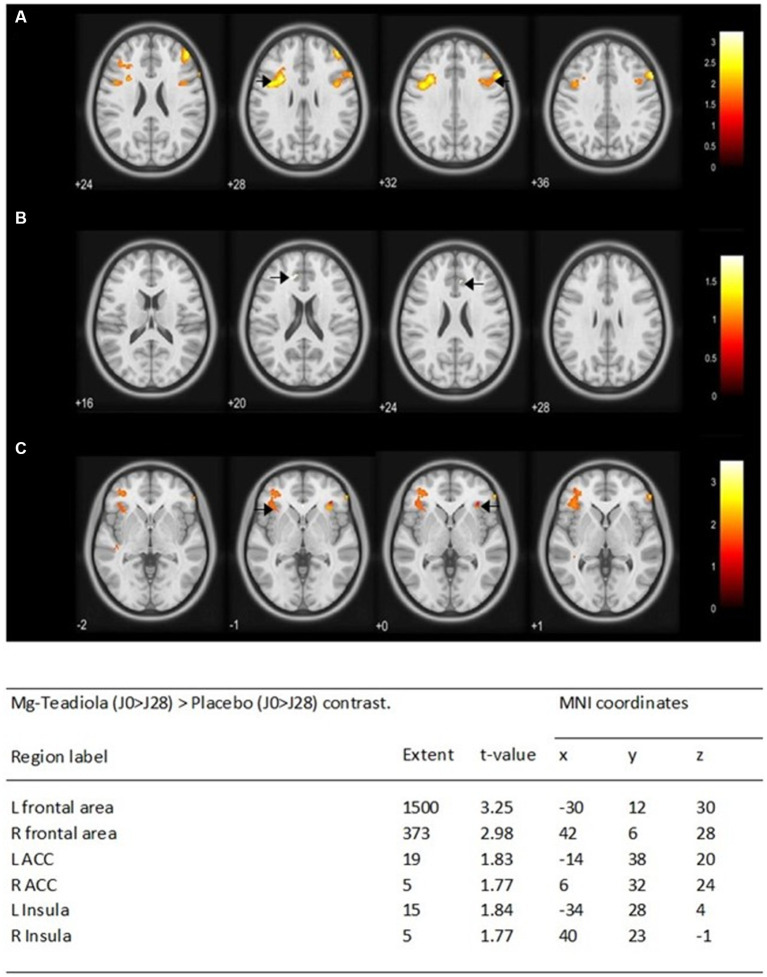
Whole-brain analysis of the blood-oxygen-level-dependent (BOLD) responses of the Mg-Teadiola (D0 > D28) > Placebo (D0 > D28) contrast during stress stimulation Statistical *t* maps were overlaid on MNI slices using a voxel-wise threshold of *p* < 0.05 and an extent threshold of five voxels. They were covering frontal area **(A)**, ACC **(B)**, and insula **(C)**. Black arrow points each region of interest at L and R. Regions were automatically labeled using the Anatomy Toolbox atlas of SPM and were presented in the table below. *x*, *y*, and *z* were MNI coordinates in the L-R, anterior–posterior, and inferior–superior dimensions, respectively. All peaks were significant at a voxel-wise threshold of *p* < 0.05 (extend threshold = 5 voxels). ACC, anterior cingulate cortex; L, left; R, right; MNI, Montreal Neurological Institute; SPM, Statistical Parametric Mapping.

Comparison of BOLD signal variations between D0 and D28 (D0 > D28) during stress stimulation between Mg-Teadiola versus placebo groups showed significantly increased activations in the left and right frontal area (*p* = 0.001 and *p* = 0.002, respectively), the left and right ACC (*p* = 0.035 and *p* = 0.04, respectively), and the left and right insula (*p* = 0.034 and *p* = 0.0402, respectively) (Figure 5).

**Figure 6:**
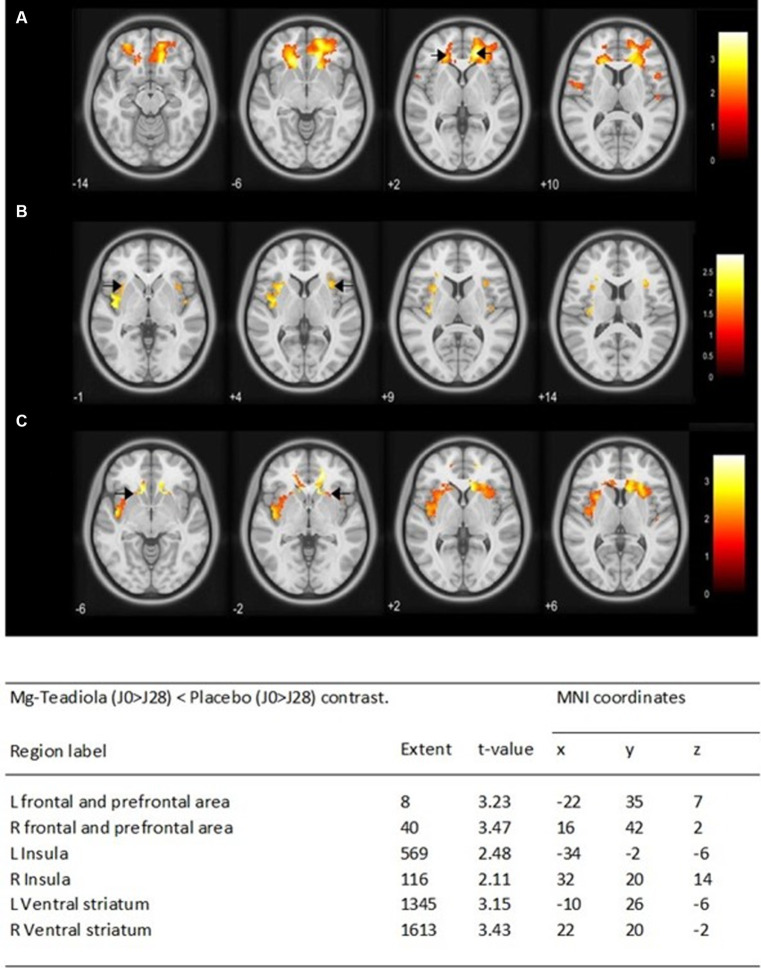
Whole-brain analysis of the BOLD responses of the Mg-Teadiola (D0 > D28) < Placebo (D0 > D28) contrast during pain stimulation Statistical *t* maps are overlaid on MNI slices using a voxel-wise threshold of *p* < 0.05 and an extent threshold of five voxels. They are covering frontal and prefrontal area **(A)**, insula **(B)**, and ventral striatum **(C)**. Black arrow points each region of interest at L and R. Regions are automatically labeled using the Anatomy Toolbox atlas of SPM and are presented in the table below. *x*, *y*, and *z* are MNI coordinates in the L-R, anterior–posterior, and inferior–superior dimensions, respectively. All peaks are significant at a voxel-wise threshold of *p* < 0.05 (extend threshold = 5 voxels). L, left; R, right; MNI, Montreal Neurological Institute; SPM, Statistical Parametric Mapping.

Comparison of BOLD signal variations between D0 and D28 (D0 > D28) during thermal pain stimulation between Mg-Teadiola and placebo groups showed significant diminished activations, mainly in the left and right prefrontal area (*p* = 0.001 and *p* = 0.001, respectively), the left and right insula (*p* = 0.008 and *p* = 0.019, respectively), and the left and right ventral striatum (*p* = 0.001 and *p* = 0.001, respectively; Figure 6).

### 4.3. Clinical outcomes

DASS-42 stress scores significantly decreased from D0 to D28 in both groups (Table 1) with a difference of five points between placebo versus Mg-Teadiola and a significant diminution was observed for Mg-Teadiola versus placebo at D14 (ES −0.60; *p* = 0.011) (Supplementary Table S1). One month after the end of treatment (D56), scores and difference between the groups remained stable (ES −0.62; *p* = 0.008) (Supplementary Table S1). A trend to diminution was also noted between D0 and D28 for anxiety and depression (Table 1). Concerning QST, sensitivity to cold improved from D0 to D28 for Mg-Teadiola versus placebo (ES 0.47; *p* = 0.042). No difference was observed for warm sensibility, warm pain, and cold pain (Table 1).

At D0, the plasma concentration of Mg was 0.85 ± 0.06 mmol/L for placebo and 0.86 ± 0.09 mmol/L for Mg-Teadiola. These measures were stable from D0 to D14 and up to D28. Urinary Mg concentration was 3.45 ± 1.49 mmol/24 h for placebo and 3.89 ± 1.58 mmol/24 h for Mg-Teadiola at D0 and remained stable on D14, D28, and D56. Cortisol was not significantly different between the groups (Table 1 and Supplementary Table S1).

Intake of Mg-Teadiola did not improve the PCS catastrophism global score from D0 to D28. However, magnification sub-scale score was significantly diminished for Mg-Teadiola at D28 (ES −0.60; *p* = 0.011) (Table 1). The same trend was observed at D14 and D56 but results did not reach statistical significance (Supplementary Table S1).

No significant difference was observed between the Mg-Teadiola and placebo groups for PSQI score between D0 and D28 (Table 1). However, subjective sleep quality (component 1) and daytime dysfunction (component 7) significantly improved for Mg-Teadiola at D14 (ES −0.47; *p* = 0.042) and D56 (ES −0.76; *p* = 0.002), respectively (Supplementary Table S1).

### 4.4. Safety outcomes

No serious AEs were reported during the study for Mg-Teadiola and placebo groups. A total of 49 non-serious AEs in the Mg-Teadiola group and 44 AEs in the placebo group were reported. A total of 10 individuals on 20 per group reported headaches or migraines, making it the highest frequency AEs reported. The second most frequently reported AE was the gastrointestinal disorder, with 7 persons on 20 per group. However, none of the AEs were considered treatment related.

## 5. Discussion

This ancillary study is the first to investigate the effect of Mg in combination with green tea, Rhodiola, and B vitamins on stress and acute pain at cerebral level, using fMRI, in chronically stressed but otherwise healthy individuals. The fMRI analysis allowed the visualization of interplay between stress and pain cerebral matrices, using thermal stimulation model to stress the system. The fMRI confirmed that supplementation with Mg-Teadiola for 28 days modulates both matrices (when stimulated) versus placebo. These results reinforce the clinical observations, showing a benefit of Mg-Teadiola on stress and pain perception.

In this study, after D28, Mg-Teadiola increased the activation of frontal cortex (FC)/PFC, AI, and ACC brain areas during the stress sequence (anticipation), while reducing the BOLD signal in FC/PFC and AI (and ventral striatum) during the thermal stimulus. Few studies have noted the significant role played by insula in processing noxious and innocuous thermal stimuli in the brain (46) and the ACC, which plays an essential role in integration of neuronal circuitry for affect regulation as it is connected to both the “emotional” limbic system and the “cognitive” PFC (47). The PFC—which has a vital role in executive functions—has connections to other areas of the cerebral neocortex, hippocampus, periaqueductal gray (PAG), thalamus, amygdala, and basal nuclei; thus, it also plays a significant role in pain processing (48). Previous studies have demonstrated the activation of PFC, AI, and ACC during the anticipation of pain using psychophysiological measures (49) and fMRI (50–52). The anticipation of pain may have an important protective function, allowing the avoidance of bodily harm through the initiation of adaptive behavior. The ACC, PFC, and insula with their associated subcortical regions, together contribute to support the mental representation of an impending stimulus (53).

Previous studies have reported that the chronic stress and stress-related conditions alter brain areas. A metanalysis reported that during depression or a post-traumatic stress disorder (PTSD), there is a reduced activation in response to emotional stimuli in the ACC (47). Across a range of psychiatric disorders, an abnormality in the structure and function of insula is observed (54), whereas individuals with a chronic back pain showed a decrease in the neural activity after the painful stimulation (55). Review articles have reported that a chronic exposure to the psychological stress stimulus may cause dysregulation in the neural pathways that are activated by dopamine, adrenaline, and cortisol and downregulation in the glucocorticoid receptor sensitivity of the PFC. Chronic stress causing the hyperactivity in the limbic system also downregulates excitatory synaptic transmission, with diminution of neural firing in the PFC and in between PFC and other cerebral neural networks having impairment of executive functioning. This further results in poor control and poor regulation over the stress responses and pain experiences, respectively (24, 56, 57).

Thus, the increased activity induced by Mg-Teadiola in these three major brain areas most probably contributed to improvement/normalization of function in the stressed population in the anticipation period. Interestingly, Boyle et al. (22) showed the capacity of Mg-Teadiola to activate the frontal theta electroencephalography (EEG) activity during cognitive tasks conducted under stressful situation (22). This observation, suggesting a capacity of the product to improve executive functions in stressed individuals under acute stressful conditions, supports the findings of the present study (22). Furthermore, because neurons in the PFC (24) and insular cortex (58) contribute to the subjective feeling of pain, the fact that Mg-Teadiola downregulated the BOLD signal in these two areas during the thermal stimulus probably helps to take away the unpleasant feeling associated with a painful event (58). This overall beneficial effect observed after acute stimulation are consistent with the favorable effects of Mg-Teadiola observed on chronic stress and sensitivity to pain during the QST, after D28.

Initially adaptive to direct brain systems for effective coping, sustained activation of the HPA axis during chronic stress can lead to a maladaptive response with the onset of chronic pain (59), mood, sleep, and cognitive functions disorders (60) and thus significantly reducing quality of life (QoL). In this study conducted in a subgroup of 40 individuals, Mg-Teadiola confirmed its potential to improve the overall QoL of chronically stressed individuals, resulting in significant decrease in DASS stress score (ES 0.46 [−0.91; −0.01], *p* = 0.048) from D0 to D28, showed a trend to improve sleep quality (at D14, Supplementary data), and decreased magnification (as tendency to increase perceived threat) in the PCS test. It also showed a reduction in sensibility to the cold pain (ES 0.47 [95% CI: 0.02; 0.92], *p* = 0.042), confirming the findings of the primary study (on 100 individuals) where a significant reduction in sensitivity to cold pain (*p* = 0.01) and a trend for lower sensitivity to warm pain (*p* = 0.06) were observed on D28. Human imaging and animal studies have shown that major brain regions (including hippocampus, amygdala, and ACC) are altered by stress (61), with chronic stress or depression often inducing synaptic plasticity as evidenced by altered glutamatergic (excitatory) and gamma-aminobutyric acid (GABA)-ergic (inhibitory) transmission (62). These two important regulatory systems are also modulated during chronic pain: animal models, such as peripheral inflammation and nerve injury produce short-term and long-term synaptic plasticity on glutamatergic transmission (63, 64), whereas GABAergic transmission is reduced in chronic pain models (65, 66). In chronic neuropathic pain individuals, magnetic resonance spectroscopy showed reduced thalamic GABA content, correlating with the degree of functional connectivity between the thalamus and the cerebral cortex, including the insula, suggesting increased activity of insula (and pain perception) (67). The observed effects of Mg-Teadiola may be explained by modulation of these two pathways by the active ingredients. Previous studies have reported that Mg blocks the N-methyl-D-aspartate (NMDA) receptor directly by inhibiting glutamate or indirectly by its enhanced reuptake into the synaptic vesicles through stimulation of the sodium–potassium P-type adenosine triphosphatase (ATPase). However, the mechanism of Mg by which it exerts a GABA-agonistic activity is not well understood (1). Mg has an important role in preventing central sensitization and attenuation of the pain hypersensitivity through its voltage-gated action at NMDA receptors and has been investigated for its ability to reduce acute or chronic pain in various clinical conditions (68). A recent study also reported a significant (*p* = 0.003) reduction in mild/moderate stress (DASS-42 stress score) and the intensity of pain (*p* = 0.029) following supplementation with oral Mg in fibromyalgia individuals (69). The important effects of the botanical extracts contained in Mg-Teadiola on brain functions have been highlighted in the previous study (20). Concerning Rhodiola, clinical data suggest that its beneficial stress-protective activity may be associated with the HPA axis and regulation of key mediators of stress response, including cortisol (70). However, the effects of Mg-Teadiola on chronic stress and related outcomes in the present study were elicited in the absence of any consistent activation of physiological markers of HPA overstimulation (e.g., cortisol). This may be related to the variability of cortisol pulsatility along the day and explain why there is often a weak correspondence between psychophysiological stress and cortisol circulating levels (71). Other hypotheses, based on preclinical studies (72, 73) suggested alterations in serotoninergic and dopaminergic activities. Regarding L-theanine—the main active constituent of green tea—neurochemistry studies have shown potential NMDA receptors modulatory effects, as well as possible increasing effects on brain serotonin, dopamine, and GABA levels (74–77) that may explain its beneficial effects on the observed outcomes. In addition, green tea extracts may contain polyphenolic compounds at significant levels (>30%) (78). Few polyphenols (mainly Epigallocatechin gallate) have been reported to potentially alter cognitive functions and mood (79). There are almost no studies reporting their effects on stress, anxiety, and depression in contrast to L-theanine (78). As the quantity of green tea extract in the product is low and a significant proportion is L-theanine, polyphenols are unlikely to play a major role in the observed results. In this study, the cognitive effect of polyphenols was not assessed separately and a synergistic effect with Mg-Teadiola could not be determined. The results obtained in this subgroup analysis involving 40 individuals confirmed the results obtained in the primary analysis that involved 100 individuals. Our study showed consistency in the fMRI data (acute stimulation) that confirmed the basal observations of improvement in the stress and pain responses. However, the study has limitations. The study observed no variations in the HPA biomarkers. Thus, further studies involving a specific design that results in an improved quality of data by involving dynamic repeated measurements of stress biomarkers upon stimulation are required. Finally, the clinical benefits of the supplementation over the stress were observed after one intake or 2 weeks; however, the fMRI experiment was conducted only after D28 of supplementation. Also, further research is required to explore and evaluate acute effects on brain matrices.

## 6. Conclusion

This fMRI study demonstrated that Mg-Teadiola, a combination of Mg, B vitamins, Rhodiola, and green tea (L-theanine) was effective in significantly modulating the brain response to stressful (thermal) stimulus in chronically stressed individuals and potentially alter the pain perception after 28 days. This response to acute stimulus was consistent with the perceived clinical long-term benefits of the product on basal stress level, pain sensitivity, and other aspects related to QoL. Data suggest a potential mechanism of action related to improved executive functions—potentially impaired by chronic stress—resulting in better control of stress response and pain experience, culminating in enhanced well-being. Further studies are needed to validate this hypothesis and further explore the mechanism of action of this promising combination of ingredients.

## Data availability statement

The raw data supporting the conclusions of this article will be made available by the authors, without undue reservation.

## Ethics statement

The study has been approved on 11 March 2020 by the Ethics committee (Sud-Est II, France), registered at the French National Agency for the Safety Medicines and Healthcare Products (ANSM) in March 2020 (2020-A00040-39) and in May 2020 at (http://www.clinicaltrials.gov). The patients/participants provided their written informed consent to participate in this study.

## Author contributions

GP and LN: conceptualization, investigation, and supervision. GP, NM, VR, and BP: methodology, resources, and visualization. VR and NM: software. GP, LN, BP, JG, VL, AT, NM, LB, CD, VR, and CC: validation and writing–review and editing. NM, BP, and CC: data curation. LN, GP, and CC: writing–original draft preparation. GP and LN: project administration and funding acquisition. All authors contributed to the article and approved the submitted version.

## Funding

This collaboration study with Sanofi was conducted by the University Hospital of Clermont-Ferrand, France. Sanofi provided the treatments and partial financial support to the study.

## Conflict of interest

LN was employed by the company Sanofi S.A.

The authors declare that this study received funding from Sanofi. The funder had the following involvement in the study: conceptualization, investigation, supervision and writing the original draft, review and editing.

## Publisher’s note

All claims expressed in this article are solely those of the authors and do not necessarily represent those of their affiliated organizations, or those of the publisher, the editors and the reviewers. Any product that may be evaluated in this article, or claim that may be made by its manufacturer, is not guaranteed or endorsed by the publisher.
