# Supplementary material for: Assessing brain function in stressed healthy individuals following the use of a combination of green tea, Rhodiola, magnesium, and B vitamins: an fMRI study
Source: Front Nutr. 2023 Aug 16;10:1211321. doi: 10.3389/fnut.2023.1211321 (PMC10469327; doi:10.3389/fnut.2023.1211321)
Supplement: Supplementary file 1 [file Data_Sheet_1.docx]

Supplementary Material

Assessing Brain Function in Stressed Healthy Individuals Following the Use of a Combination of Green Tea, Rhodiola, Magnesium, and B Vitamins: An fMRI Study

Gisèle Pickering, Lionel Noah, Bruno Pereira, Jonathan Goubayon, Vincent Leray, Ambre Touron, Nicolas Macian, Lise Bernard, Christian Dualé^1^, Veronique Roux, Carine Chassain

*** Correspondence:** Gisèle Pickering: gisele.pickering@uca.fr

# Supplementary Data

**Supplementary materials:** Information on inclusion and exclusion criteria

Inclusion criteria:

- Male or female aged 18–65 years,
- Individual with stress (DASS-42 screening scale ≥14) and having been under stress for at least 1 month,
- Individual free of any introduction of a new treatment or diet at the time of inclusion,
- Individual free of any treatment in the 7 days prior to inclusion, including no use of analgesic or anti-inflammatory drugs,
- Cooperation and understanding sufficient to comply with the requirements of the study,
- Acceptance to give written consent,
- Affiliation with the French Social Security,
- Registration or acceptance of registration in the national register of volunteers participating in Research.

Exclusion criteria:

- Contraindications to the realization of MRI without injection such as claustrophobia or use of a hearing aid, a pacemaker, or a brain clip,
- Contraindication to Mg administration: hypersensitivity to Mg oxide or to one of the excipients,
- With magnesemia >1.07 mmol/L,
- With moderate (or more severe) kidney failure with creatinine clearance <60 mL/min,
- Receiving a treatment or dietary supplement containing Mg, pre- or probiotics, or herbal extracts (e.g., antistress, anti-inflammatory, or analgesic) at the time of inclusion,
- Treated with antibiotics in the 3 months prior to inclusion,
- Having a medical and/or surgical history judged by the investigator or his or her representative that are not compatible with the trial,
- Evolutionary pathology at the time of the inclusion,
- Excessive consumption of alcohol, tobacco (up to 10 cigarettes per day), coffee, tea, or drink containing caffeine (equivalent to more than 4 cups per day) or substance abuse,
- Individual who does not meet the selection criteria for their ability to discriminate against sensations resulting from nociceptive stimulation during psychometric tests,
- Individual who does not meet the selection criteria for their ability to discriminate against colors projected during the fMRI exam,
- Individual involved in another clinical trial, or in the exclusion period, or having received a total amount of compensation of more than 4,500 euros over the 12 months prior to the start of the trial,
- Woman of childbearing age not using an effective contraceptive method, pregnant woman, or breastfeeding.

# Supplementary Tables

**Supplementary Table 1.** DASS-42, PCS-catastrophizing, PSQI-sleep, and biology measures, following supplementation with Mg-Teadiola versus placebo at visit 2 (D0), visit 3 (day 14), and visit 5 (day 56)

|  | **D0** | | **D14** | | | **D56** | | |
| --- | --- | --- | --- | --- | --- | --- | --- | --- |
|  | **Placebo**  ***n* = 20** | **Mg-Teadiola**  ***n* = 20** | **Placebo**  ***n* = 20** | **Mg-Teadiola**  ***n* = 20** | **ES (95% CI)**  ***p*-value** | **Placebo**  ***n* = 20** | **Mg-Teadiola**  ***n* = 20** | **ES (95% CI)**  ***p*-value** |
| **DASS-42** |  |  |  |  |  |  |  |  |
| DASS stress | 29.6 ± 7.8 | 24.4 ± 6.1 | 25.5 ± 8.9 | 19.9 ± 4.9 | **−0.60 [−1.05; −0.14]**  ***p* = 0.011** | 23.0 ± 9.8 | 15.7 ± 7.0 | **−0.62 [-1.08; −0.17]**  ***p* = 0.008** |
| DASS anxiety | 17.4 ± 7.8 | 12.9 ± 8.1 | 14.0 ± 7.5 | 9.8 ± 7.0 | −0.12 [−0.58; 0.33]  *p* = 0.581 | 12.0 ± 8.4 | 7.0 ± 6.3 | −0.26 [−0.71; 0.20]  *p* = 0.258 |
| DASS depression | 11.5 ± 9.4 | 9.9 ± 7.7 | 8.6 ± 8.2 | 6.7 ± 5.7 | −0.14 [−0.59; 0.31]  *p* = 0.536 | 7.5 ± 7.5 | 4.8 ± 5.2 | −0.26 [−0.72; 0.19]  *p* = 0.244 |
| **PCS-catastrophism** | 18.9 ± 11.6 | 13.3 ± 8.3 | 14.9 ± 11.0 | 11.4 ± 8.0 | 0.07 [−0.38; 0.52]  *p* = 0.757 | 14.6 ± 11.8 | 8.7 ± 7.6 | −0.19 [−0.64; 0.26]  *p* = 0.401 |
| Rumination | 6.6 ± 4.7 | 4.7 ± 3.2 | 4.5 ± 3.7 | 4.1 ± 3.4 | 0.14 [−0.32; 0.59]  *p* = 0.547 | 4.9 ± 4.2 | 2.2 ± 2.1 | −0.44 [−0.89; 0.01]  *p* = 0.055 |
| Magnification | 4.7 ± 3.2 | 4.1 ± 3.1 | 4.1 ± 2.9 | 2.8 ± 2.4 | −0.34 [−0.80; 0.11]  *p* = 0.132 | 3.8 ± 3.0 | 2.8 ± 2.8 | −0.20 [−0.65; 0.26]  *p* = 0.388 |
| Helplessness | 7.7 ± 5.8 | 4.6 ± 3.7 | 6.3 ± 5.9 | 4.5 ± 3.3 | 0.13 [−0.33; 0.58]  *p* = 0.579 | 5.9 ± 5.7 | 3.7 ± 3.4 | −0.03 [−0.48; 0.42]  *p* = 0.894 |
| **PSQI-sleep** | 8.7 ± 2.6 | 7.0 ± 2.4 | 8.2 ± 3.0 | 6.1 ± 2.2 | −0.31 [−0.77; 0.14]  *p* = 0.169 | 7.0 ± 2.6 | 5.0 ± 1.9 | −0.42 [−0.87; 0.03]  *p* = 0.069 |
| Subjective sleep quality | 1.8 ± 0.6 | 1.6 ± 0.7 | 1.9 ± 0.7 | 1.4 ± 0.5 | **−0.47 [−0.92; −0.02]**  ***p* = 0.042** | 1.4 ± 0.6 | 1.3 ± 0.6 | −0.15 [−0.60; 0.30]  *p* = 0.503 |
| Sleep latency | 2.2 ± 1.0 | 2.0 ± 0.9 | 2.0 ± 1.1 | 1.6 ± 1.0 | −0.14 [−0.60; 0.31]  *p* = 0.527 | 1.9 ± 0.9 | 1.5 ± 0.8 | −0.31 [−0.76; 0.15]  *p* = 0.177 |
| Sleep duration | 0.7 ± 0.9 | 0.6 ± 0.8 | 0.8 ± 0.9 | 0.6 ± 0.8 | −0.23 [−0.68; 0.22]  *p* = 0.307 | 0.7 ± 0.7 | 0.5 ± 0.8 | −0.19 [−0.64; 0.26]  *p* = 0.405 |
| Habitual sleep efficiency | 0.5 ± 0.8 | 0.2 ± 0.4 | 0.6 ± 0.9 | 0.2 ± 0.4 | −0.26 [−0.71; 0.20]  *p* = 0.260 | 0.3 ± 0.6 | 0.1 ± 0.3 | −0.10 [−0.56; 0.35]  *p* = 0.642 |
| Sleep disturbances | 1.7 ± 0.5 | 1.3 ± 0.4 | 1.4 ± 0.5 | 1.1 ± 0.3 | −0.18 [−0.64; 0.27]  *p* = 0.415 | 1.4 ± 0.5 | 1.1 ± 0.3 | −0.25 [−0.71; 0.20]  *p* = 0.265 |
| Use of sleeping medication | 0.3 ± 0.8 | 0.1 ± 0.3 | 0.3 ± 0.7 | 0.0 ± 0.0 | −0.38 [−0.83; 0.08]  *p* = 0.101 | 0.2 ± 0.7 | 0.1 ± 0.2 | −0.11 [−0.57;0.34]  *p* = 0.615 |
| Daytime dysfunction | 1.6 ± 0.7 | 1.3 ± 0.5 | 1.3 ± 0.7 | 1.2 ± 0.4 | 0.15 [−0.30; 0.60]  *p* = 0.510 | 1.3 ± 0.6 | 0.6 ± 0.5 | **−0.76 [−1.21; −0.30]**  ***p* = 0.002** |
| **Biology measures** |  |  |  | | |  | | |
| Plasma Mg (mmol/L) | 0.85 ± 0.06 | 0.86 ± 0.09 | 0.85 ± 0.05 | 0.88 ± 0.06 | 0.44 [−0.19; 1.09]  *p* = 0.171 | 0.85 ± 0.05 | 0.89 ± 0.07 | 0.61 [−0.02; 1.25]  *p* = 0.057 |
| Urinary Mg (mmol/24h) | 3.45 ± 1.49 | 3.89 ± 1.58 | 3.36 ± 1.22 | 3.94 ± 1.37 | 0.44 [−0.20; 1.06]  *p* = 0.178 | 3.24 ± 1.61 | 3.17 ± 1.02 | −0.05 [−0.67; 0.56]  *p* = 0.870 |

Values are mean ± SD unless otherwise stated. Significant *p*-values are in bold.

CI, confidence interval; D, day; DASS, Depression Anxiety Stress Scale; ES, effect size; Mg, magnesium; PCS, Pain Catastrophizing Scale; PSQI, Pittsburgh Sleep Quality Index
